# HOXC6 overexpression stimulates cell migration and correlates with poor prognosis in head and neck squamous cell carcinoma

**DOI:** 10.1007/s00018-025-06039-3

**Published:** 2026-01-11

**Authors:** Subhendu Roy Choudhury, Ishita Gupta, Ian Mills, Vera Mukhina, Andrey Loginov, Erin Allor, Alexa Anderson, Ashley Cellini, Carol Robles, Donita Dyalram, Joshua Lubek, Jeffrey Wolf, Rodney Taylor, Kyle Hatten, Nadezhda Vorobyeva, Daria A. Gaykalova

**Affiliations:** 1 Institute for Genome Sciences, University of Maryland School of Medicine, Baltimore, MD USA; 2 Department of Otorhinolaryngology-Head and Neck Surgery, Marlene & Stewart Greenebaum Comprehensive Cancer Center, University of Maryland Medical Center, Baltimore, MD USA; 3 University of Maryland School of Medicine, Baltimore, MD USA; 4 Institute of Gene Biology, Russian Academy of Sciences, Moscow, Russia; 5 Department of Oncology, Sidney Kimmel Comprehensive Cancer Center, Johns Hopkins University, Baltimore, MD USA; 6 Department of Cancer Genetics, Institute for Cancer Research, Oslo University Hospital, Oslo, Norway; 7 Vavilov Institute of General Genetics, Russian Academy of Sciences, Moscow, Russia; 8 Institute for Genome Sciences, 670 West Baltimore Street, Baltimore, MD 21201 USA

**Keywords:** HOXC6, HNSCC, Oncogene, Biomarker

## Abstract

Head and neck squamous cell carcinoma (HNSCC) is the sixth most common cancer worldwide and accounts for 2% of cancer-related deaths. Although the oncogenic role of HOXC6 in solid tumors is known, its functional relevance in HNSCC remains elusive. Using The Cancer Genome Atlas (TCGA, with 520 HNSCC samples) data and in vitro models, we investigated the functional role of HOXC6 in HNSCC. TCGA analysis revealed that HOXC6 overexpression in HNSCC tissues correlated with malignant progression and poor survival outcomes. In vitro studies confirmed HOXC6 overexpression in HNSCC cell lines, and knockdown of HOXC6 significantly reduced both cell proliferation and migration, highlighting the oncogenic role of HOXC6. Additionally, pathway analysis of RNA-seq data linked HOXC6 expression with immune evasion and dysregulation of cell cycle genes, particularly the E2F and G2M checkpoints. Furthermore, H3K27ac ChIP-seq data showed that histone acetylation at the HOXC6 promoter drives HOXC6 overexpression. This study identified HOXC6 as a key oncogenic driver in HNSCC and as a candidate biomarker for HNSCC. Targeting HOXC6 could pave the way for improved biomarker-driven approaches in HNSCC treatment to reduce recurrence and improve patient survival rates.

**Supplementary Information:**

The online version contains supplementary material available at 10.1007/s00018-025-06039-3.

## Introduction

Head and neck squamous cell carcinoma (HNSCC) represents a heterogeneous group of cancers originating from squamous cells lining the mucosal surfaces of the oral cavity, larynx, and pharynx [1]. Globally, HNSCC is the seventh most common cancer with various etiological factors, including smoking tobacco, alcohol intake, consumption of areca nut or betel leaf, and human papillomavirus (HPV) infection [2], accounting for approximately 25% of HNSCC cases [3]. Molecular alterations in HNSCC include amplification and overexpression of oncogenes (MYC, ERBB2, EGFR, and CCND1), mutations inactivating tumor suppressor genes (CDKN2A and TP53), and loss of heterozygosity, which are associated with poor prognosis in HNSCC [4]. Although the U.S. Food and Drug Administration (FDA)-approved drugs (pembrolizumab, nivolumab, and cetuximab) are available for treatment [5, 6], the low response rate to these drugs [7, 8] and the development of resistance leads to treatment failure, tumor relapse, and metastasis. One plausible reason for resistance to immunotherapy could be the presence of an immunosuppressive tumor microenvironment (TME) comprised of regulatory T-cells, macrophages, infiltrating immune cells, and fibroblasts [9]. Additionally, epithelial-to-mesenchymal transition (EMT) and an altered cytokine landscape (elevated TGF-β and IL-10 levels) contribute to immune evasion, with tumor heterogeneity further influencing response rates [9]. Identifying novel therapeutic targets to understand the molecular mechanisms underlying the onset and progression of HNSCC is crucial for improving patient outcomes.

Our preliminary analysis revealed that HOXC6 is associated with poor overall survival and lymph node metastasis, highlighting its potential as an oncogenic candidate, warranting further investigation. HOXC6 belongs to the HOX family of transcription factors that regulate embryonic development and several cellular events, such as differentiation and morphogenesis [10] through the regulation of its targets (BMP7, FGFR2, and PDGFRA) [11] and signaling pathways (TGF-β/SMAD, PI3K/Akt, MAPK, Notch, and Wnt) [12–15]. Deregulation of HOXC6 is associated with the onset of various types of human cancers, such as glioblastoma, prostate cancer, and colorectal cancer [15–17]. Likewise, in esophageal squamous cell carcinoma [18] and HNSCC, elevated HOXC6 expression correlates with increased Bcl-2 levels, suggesting a role for HOXC6 in inhibiting apoptosis and promoting cell growth [19]. However, the precise function of HOXC6 in HNSCC remains unclear.

In this study, we obtained clinical and expression data for HNSCC from TCGA to analyze HOXC6 expression and prognostic significance in patients with HNSCC using Kaplan-Meier survival analysis. RNA-sequencing and ChIP-sequencing data were used to investigate transcriptional regulation, while Gene Set Enrichment Analysis (GSEA) was performed to identify associated pathways. Additionally, functional assays were performed to explore the role of HOXC6 in HNSCC progression. Our data demonstrate that HOXC6 acts as an oncogenic driver in HNSCC progression, and its upregulation in tumor samples appears to be associated with increased promoter acetylation.

## Methods

### TCGA patient cohort and survival analysis

Clinical data for HNSCC were obtained from TCGA via the cBio Cancer Genomics Portal (http://cbioportal.org) (RRID: SCR_014555) [20, 21] and downloaded using TCGAbiolinks R package (RRID: SCR_017683) [22, 23]. In total, 530 patients with HNSCC were included. Samples with missing HPV status, duplicated entries, and missing survival data were excluded, yielding a final analytical cohort of 519 tumor samples. In addition, 44 normal tissue samples were used as the controls. Clinical parameters, including patient age, sex, tumor stage, smoking status, and survival data, were extracted for the correlation studies. Of the 519 TCGA-HNSCC samples, 136 (26.2%) were females and 383 (73.85) were males, with an average patient age of 60.87 years (*±* 11.87 SD).

Differential HOXC6 expression levels between normal tissues and HNSCC samples were obtained from the Gene Expression Profiling Interactive Analysis (GEPIA) database (RRID: SCR_018294) [24] and analyzed using the Wilcoxon rank-sum. We also analyzed HOXC6 expression in a gene expression microarray cohort (GSE33205) [25] compared to uvulopalatopharyngoplasty (UPPP) controls using Welch’s t-test. The HOXC6 expression among lymph node stages (N) was analyzed in the GEPIA dataset (RRID: SCR_018294) using the Kruskal-Wallis test, followed by Dunn’s test with Benjamini-Hochberg adjustment for multiple comparisons. The comparison of HOXC6 expression between M0 and M1 metastasis stages was performed using the Wilcoxon rank-sum test. Kaplan-Meier curves were generated to estimate the probability of survival over time for the entire TCGA HNSCC cohort. Survival data for one patient were missing, resulting in a final cohort of 519 tumor samples. Based on the median transcriptomic expression of HOXC6 (2.109 transcripts per million (TPM)), patients were classified into “low” and “high” HOXC6 expression, and the overall survival (OS) was analyzed. Survival rates were compared using the Kaplan-Meier method with the log-rank test.

### UMB sample collection and processing

Primary tumor tissue samples were collected from a cohort of 10 patients with head and neck squamous cell carcinoma and healthy mucosal tissue from the same patients from the University of Maryland, Baltimore (UMB), after surgical resection procedures. All tissue samples were obtained with approval from the institutional review board (IRB) protocol (IRB# HP-00095899). This study was exempted from the U.S. Department of Health and Human Services policy for the protection of human subjects (IRB# HP-00095899).

For sample processing, unwanted materials such as fat and necrotic tissue were removed from the sample. The tissue was snap-frozen in liquid nitrogen to preserve the nucleic acid integrity for downstream analysis. For downstream processing, frozen tissue was thawed on ice to prevent degradation. Following thawing, the tissue was minced into smaller pieces and homogenized for RNA extraction. The processed samples were stored at −80 °C.

### Cell culture

The normal cell lines HOK16B (RRID: CVCL_B405) and OKF6 (RRID: CVCL_L224) were provided by Dr. No-Hee Park from the University of California, Los Angeles, and Dr. David Sidransky from Johns Hopkins University, Baltimore, respectively. The human HNSCC cell lines UMSCC-104 (RRID: CVCL_7712) and UMSCC-047 were provided by Dr. Thomas Carey of the University of Michigan. In addition, the HNSCC cell lines UPCI-SCC-090 (RRID: CVCL_1899) and 93VU-147T (RRID: CVCL_L895) were provided by Dr. Susanne M. Gollin from the University of Pittsburgh and Dr. Johan de Winter from VU University Medical Center, respectively. All cell lines used in this study were authenticated by short tandem repeat (STR) profiling and tested negative for mycoplasma contamination.

All four HNSCC cell lines were cultured in high-glucose DMEM (Invitrogen) supplemented with 10% Fetal Bovine Serum (FBS; Gemini) and 1% Penicillin and Streptomycin (P/S) (Corning). Additionally, UMSCC-104 and UMSCC-047 cells were cultured with 1% non-essential amino acids (NEAA). The non-cancerous cell lines OKF6 and HOK16B were cultured in keratinocyte serum-free media (SFM) (Invitrogen) supplemented with 1% P/S along with bovine pituitary extract (BPE) and epidermal growth factor (EGF) All cells were cultured in a humidified chamber with 5% CO_2_ at 37 °C All experiments were performed when the cells reached a confluent state of ~ 70–80%.

### HOXC6 SiRNA transfection of HNSCC cell lines

The UMSCC-047 and 93VU-147T cell lines were transiently knocked down with HOXC6-specific siRNA. siRNA was obtained from Dharmacon, utilizing ON-TARGETplus SMARTpool (L-011871-00-0005, siRNA-HOXC6) and a scrambled ON-TARGETplus non-targeting pool siRNA (D-001810-05, Control siRNA).

UMSCC-047 and 93VU-147T cells were plated and cultured in 6-well plates at densities of 1 × 10^5^ cells/well and 3 × 10^5^ cells/well, respectively, with three replicates per experiment. Upon reaching approximately 80% confluency, cells were transfected in reduced-serum medium (Opti-MEM, Gibco) with 20 nM siRNA using Lipofectamine RNAi-MAX reagent (Invitrogen) for 16 h. The transfected medium was replaced with complete DMEM with the necessary supplements (see above) after 16 h of transfection as per the manufacturer’s protocol. Cells were harvested 72 h after transient knockdown for RNA isolation. The knockdown efficiency for HOXC6 was determined using quantitative real-time PCR (qRT-PCR).

### Cell proliferation assay

The Alamar Blue Cell Viability Reagent (Bio-Rad, USA) was used to determine cell viability according to the manufacturer’s protocol [26]. The HNSCC cell lines UMSCC-047 and 93VU-147T were treated with Alamar Blue (Bio-Rad) diluted 1:10 in Opti-MEM Media (Gibco) and incubated for two hours at 37 °C in a 5% CO_2_ atmosphere protected from light. Fluorescence intensity was measured at 0 h (before transfection) to establish baseline viability at an excitation/emission wavelength of 530/590 nm on a Spectramax M5 microplate reader. The cells were then transfected with the respective siRNAs, as described above. After transfection, Alamar Blue reagent was added to each well, and fluorescence intensity was measured at 24, 48, 72, and 96 h after transfection.

### Wound healing assay

A wound-healing assay was performed to evaluate the migratory ability of cells upon siRNA-mediated HOXC6 knockdown. Briefly, adherent cells plated for transfection in 6-well plates (see above) were washed with PBS. Following a PBS wash, a sterile 200-µl pipette tip was used to create a uniform scratch vertically through the middle of each well. Detached cells were removed by washing with PBS before adding DMEM. Images were captured 0 h (before transfection) to establish the baseline wound area. The cells were then transfected with the respective siRNAs, as described above. Wound closure was monitored and imaged at 24, 48, and 72-hours post-transfection until the wound healed/closed for any one condition using the EVOS M7000 Imaging System (Invitrogen) until the wound healed or closed for any one condition. The wound area was quantified using the ImageJ software (RRID: SCR_003070) [27], and the percentage of wound closure was calculated relative to the initial wound area. Each experiment was performed in triplicates to ensure reproducibility.

### Quantitative real-time PCR (qRT-PCR)

For gene expression analysis, approximately 1 × 10^6^ cells from cell lines (UMSCC-47, UPCI-SSC-O90, 93VU-147T, HOK16B, and OKF6) and a small piece of tissue sample were used for total RNA extraction using the mirVana miRNA Isolation Kit (Ambion, Foster City, CA, USA) according to the manufacturer’s instructions. RNA concentration was quantified using a NanoDrop ND-1000 spectrophotometer (NanoDrop Technologies, Inc.). Samples were required to achieve an RNA Integrity Number (RIN) of at least 7.0. Reverse Transcription was performed using the Reverse Transcription MultiScribe™ Reverse Transcription kit (Invitrogen) with 1 µg of RNA as a template for cell lines and 500 ng for tissue samples.

qRT-PCR was performed using TaqMan Gene Expression Assays (Applied Biosystems) to quantify the expression of HOXC6 (Hs00171690_m1) relative to the housekeeping control, ACTB (Hs01060665_g1). Each qRT-PCR reaction consisted of 10 µL TaqMan Universal PCR Master Mix, 1 µL TaqMan probe, and 8 ng cDNA template. The reactions were performed on a quantitative real-time PCR System (Bio-Rad CFX 384), according to the manufacturer’s recommendations. Each sample was analyzed in triplicate and underwent one cycle of 10 min at 95 °C, followed by 50 cycles of 15 s at 95 °C, and 60 s at 60 °C. Relative fold enrichment was quantified in triplicate relative to the ACTB sample using the ΔΔCt method [28].

### RNA-sequencing

Maryland Genomics (MDG) at the Institute for Genome Sciences (IGS), University of Maryland School of Medicine (UMSOM), performed RNA sequencing. For RNA-sequencing, 280 ng RNA was used for library preparation. Strand-specific, dual-unique indexed libraries for sequencing were prepared using the NEBNext^®^ Ultra™ II Directional RNA Library Prep Kit for Illumina^®^ (New England Biolabs, Ipswich, MA). Library size selection was performed using SPRIselect beads (Beckman Coulter Genomics, Danvers, MA, USA). NovaSeq 6000 (Illumina) was used for library sequencing.

### RNA-seq data processing and analysis

The cell lines and patient samples were analyzed separately. As only one replicate of the OKF6 cell line RNA-seq was sequenced along with other cell lines, we added three more replicates of OKF6 RNA-seq data from the SRA (bioproject PRJNA322491, untreated samples). Nextflow rnaseq pipeline v.3.14.0 [29]. The STAR + Salmon branch was used to process and map reads onto an hg38 reference genome and obtain counts.

RNA sequencing data were normalized based on version 2 protocols developed by TCGA [30]. Differential expression analysis was conducted using the DeSeq2 R package (RRID: SCR_015687) [31] with the Wald test, applying thresholds of |LFC| >1 and an adjusted p-value < 0.05. For patient samples, five tumor samples with the highest HOXC6 expression were compared to five tumors with the lowest HOXC6 expression. For the siRNA-mediated HOXC6 knockdown experiment, scrambled siRNA control samples were compared with the siRNA-HOXC6 samples.

### Chromatin Immunoprecipitation (ChIP)

ChIP-DNA was prepared using the SimpleChIP Enzymatic Chromatin Immunoprecipitation (IP) Kit #9005 (Cell Signaling Technology), following the manufacturer’s protocol. We followed the sonication steps for chromatin preparation with sample-specific adjustments and omitted the MNase digestion steps. We used 25–50 mg of primary tissue for chromatin preparation, and 4 × 10^6^ cells for each ChIP cell line. For tissue samples, immunoprecipitation (IP) was performed with 5-10ug chromatin and for cell lines with 10ug chromatin with the acetyl-histone H3 Lys27 (H3K27ac) rabbit monoclonal antibody (Cat# 8173, Cell Signaling Technology) (RRID: AB_10949503) at a 1:50 dilution or with negative control Normal Rabbit IgG (Cat# 2729, Cell Signaling Technology) (RRID: AB_1031062) at a 1:250 dilution. ChIP-DNA was purified and measured using the nanodrop.

To assess BRD4 occupancy at the HOXC6 promoter in normal oral keratinocyte (OKF6) and the HNSCC (UMSCC-47) cell lines, cells were cross-linked with 1% formaldehyde for 30 min at room temperature and quenched with 125mM glycine. Nuclei were isolated and chromatin was sheared by sonication to generate DNA fragments of approximately 200–500 bp. IPs was performed with 5ug chromatin with the anti-BRD4 antibody (E2A7X) rabbit monoclonal antibody (Cat# 13440 Cell Signaling Technology) (RRID: AB_2687578) at a 1:50 dilution, the histone H3 (D2B12) XP rabbit monoclonal antibody (Cat# 4620, Cell Signaling Technology) (RRID: AB_1904005) at a 1:50 dilution or with negative control Normal Rabbit IgG (Cat# 2729, Cell Signaling Technology) (RRID: AB_1031062) at a 1:250 dilution. ChIP-DNA was purified and measured using the nanodrop.

### ChIP-DNA-based qPCR

ChIP-DNA-based quantitative PCR was performed using a TaqMan quantitative real-time PCR System (Bio-Rad CFX 384), according to the manufacturer’s recommendations. The Johns Hopkins lab standard 10× PCR Buffer [32], 0.6% Platinum^®^ Taq DNA Polymerase (Invitrogen), 2% ROX Reference Dye (Invitrogen), 0.2 mM of dNTPs (Bioline), 0.6 µM of each primer, and 0.33 µM of probe (Thermo Fisher Scientific) were used. Primers and probes were designed for the promoter region of actively expressed GAPDH, the 3′ end of the transcriptionally repressed ZNF333 gene [33], and the HOXC6 promoter region (Supplementary Table S1). ChIP DNA (5-10ng) from each sample was analyzed in duplicate and subjected to one cycle of 10 min at 95 °C, followed by 50 cycles of 15 s at 95 °C and 60 s at 60 °C. Relative fold enrichment was quantified in duplicate relative to the 2% input sample using the 2 − ΔΔCt method [28].

### ChIP-sequencing

MDG at the IGS performed ChIP sequencing. For ChIP-Seq, we used the KAPA Hyper Prep Kit for Illumina^®^ platforms to prepare sequencing libraries, following the manufacturer’s protocol. Briefly, ChIP DNA samples (0.5ng) were subjected to end repair and A-tailing in a single reaction using KAPA End Repair & A-Tailing Buffer and Enzyme Mix. Adapter ligation was performed with Illumina-compatible adapters, followed by a 0.8X SPRI^®^ bead cleanup to remove excess adapters and adapter dimers. The adapter-ligated DNA was then amplified using KAPA HiFi HotStart ReadyMix for high-fidelity library amplification, and the number of PCR cycles was optimized to prevent over-amplification. The final libraries were purified using a 1X SPRI^®^ bead cleanup and assessed for quality and concentration using an Agilent Bioanalyzer and Qubit fluorometer, respectively. Libraries were sequenced on the Illumina platform using paired-end reads.

### ChIP-sequencing data processing

Raw sequencing reads were pre-processed using Trimmomatic (version 0.38) (RRID: SCR_011848) [34] to obtain adapter-free reads suitable for downstream analysis. In particular, Illumina adapter sequences were removed using the ILLUMINACLIP function, followed by trimming low-quality bases from both the start (LEADING:3) and end (TRAILING:3) of the reads. Sliding window trimming was applied to remove regions with an average quality score below 12 (SLIDINGWINDOW: 8:12) and reads shorter than 60 bases (MINLEN:60) were discarded. The pre-processed reads were subsequently aligned to the hg38 reference genome using the BWA-MEM2 algorithm (version 2.2.1) [35]. Coverage tracks were generated using the bamCoverage tool in the deepTools suite (version 3.5.1) [36]. Coverage computation was performed with a bin size of 100 bp (--binSize 100), and the coverage was normalized using the Reads Per Genomic Content (RPGC) method (--normalizeUsing RPGC). An adequate genome size of 2,913,022,398 bases (--effective GenomeSize 2913022398) was specified. Reads were extended to their full fragment length (extendReads), and duplicate reads were excluded to minimize bias (ignoreDuplicates).

#### RNA-seq and ChIP-seq correlation

Spearman’s correlation was used to compare the expression of a cluster of HOXC genes (by RNA-seq) located in the chr12:53935327–54,033,051 interval to the intensity of the super-enhancer found near the HOXC6 gene within the chr12:54015618–54,027,261 interval. The super-enhancer intensity for each sample was calculated as the area under the curve for normalized H3K27ac ChIP-seq coverage.

### Pathway analysis

Gene Set Enrichment Analysis (GSEA) utilizing hallmark pathways from MSigDB as reference [37] was performed using the fgsea R package [38].

### In vitro statistical analysis

qRT-PCR data were analyzed, and graphs were plotted using GraphPad Prism software (version 10) (RRID: SCR_002798). Data are shown as the mean ± standard error of the mean (SEM) from three independent experiments performed in triplicate. Paired t-tests were used to compare mRNA knockdown efficiency in siRNA-HOXC6 relative to the scrambled siRNA control.

Two-way ANOVA, followed by Sidak’s multiple comparison tests, was used to compare the differences between the scrambled siRNA control and siRNA-HOXC6 at different time points to compare cell proliferation and migration rates. Differences were considered statistically significant at *p* < 0.05.

## Results

### HOXC6 is the potential candidate to study its role in HNSCC tumorigenesis

**Fig. 1:**
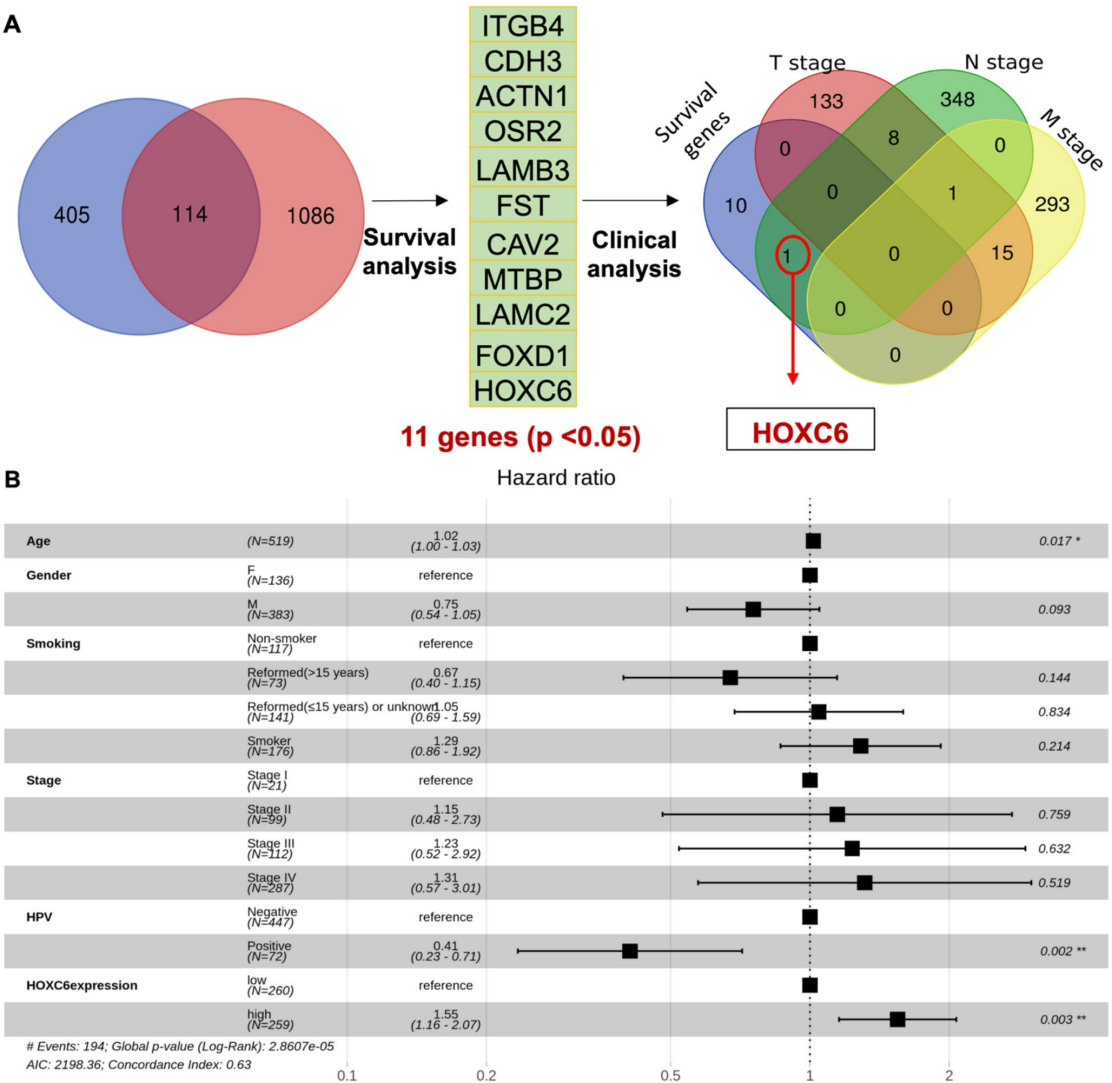
HOXC6 is a potential candidate in HNSCC carcinogenesis. (**A**)Left: Venn diagram between the genes (n=519) with HNSCC-specific H3K27ac enrichment (blue) and genes over-expressed in TCGA-HNSCC (n=1200, red), with 114 genes being common. Middle: HNSCC survival analysis for 114 common genes (by GEPIA) detected 11 genes to have a positive correlation with poor overall survival. Right: A Venn diagram between the 11 survival genes and genes that have a significant correlation (p-value < 0.05) with HNSCC clinical stages (TNM, T-tumor, N-lymph node, and M-metastasis). Only one gene (HOXC6), red circle and indicated by arrow, correlates with the N stage. (**B**).Forest plot of the hazard ratios (HR) and 95% confidence intervals (CI) for various clinical and molecular variables in a multivariate Cox proportional hazards model performed on the TCGA HNSCC cohort

Our previous study found that disease-specific H3K27ac enrichment was present in almost 519 genes in HNSCC models (Supplementary Table S2A) [39]. To link histone mark enrichment to expression, we compared these 519 genes with the most upregulated genes in HNSCC. We retrieved a total of 1200 genes with log2 fold > 1.098 and p-value < 0.05 from the TCGA-HNSCC dataset using the online web tool Gene Expression Profiling Interactive Analysis (GEPIA, cancer-pku.cn) [24] (Supplementary Table S2B) (Fig. 1 A). We found 114 genes common to both datasets, suggesting differential H3K27ac enrichment and differential expression. Next, we examined whether these genes were related to overall survival. Using the GEPIA online tool, out of the 114 genes, we found 11 genes (*p* < 0.05) to positively correlate with poor overall survival (Fig. 1 A).

We further assessed the clinical parameters related to TNM staging (T-tumor size, N-lymph node, and M-metastasis) for this set of 11 HNSCC genes. Using the OncoDB tool (http://oncodb.org) [40], we initially identified 157 genes for the T stage, 358 genes for the N stage, and 309 genes for the M stage (*p* < 0.05) that exhibited a positive correlation between their expression profiles and clinical parameters (Fig. 1 A) (Supplementary Table S2C). By intersecting the genes associated with each TNM stage, we found that only HOXC6 expression was correlated with the N stage (Fig. 1 A). These findings suggest that the aberrant expression of HOXC6 is clinically associated with lymph node metastasis in HNSCC.

In addition, we examined HOXC6 expression in other types of cancer using TIMER2.0 software [41], which showed upregulated levels of HOXC6 in several other cancers, including HNSCC (Supplementary Fig. 1). Based on these observations, HOXC6 was selected as an oncogenic candidate for HNSCC treatment.

To validate our hypothesis regarding the involvement of HOXC6 deregulation in HNSCC tumorigenesis, we analyzed HOXC6 expression using the TCGA HNSCC dataset and the gene expression microarray cohort (GSE33205). Analysis revealed that the HOXC6 median expression levels were significantly higher in the tumor samples (*p* < 0.05) than in the normal control samples (Supplementary Fig. 2 A and B). Furthermore, we demonstrated that in the HNSCC TCGA dataset, median HOXC6 expression slightly differed among N stages (Kruskal-Wallis, *p* = 0.01), specifically between N0 and N2 stages (Dunn, padj < 0.05) (Supplementary Fig. 2 C), indicating that overexpression of HOXC6 correlates with lymph node metastasis. Nevertheless, the positive correlation with M stage was not statistically significant, as there were only six patients within the M1 stage in the entire TCGA cohort (Supplementary Fig. 2D). Multivariate Cox analysis demonstrated that HOXC6 high expression was a good predictor of survival, along with age and HPV status (Fig. 1B). Patients with HOXC6 expression above the median expression in tumor samples (Supplementary Table S2D) showed worse survival rates than patients with lower HOXC6 expression (*p* = 0.01) (Supplementary Fig. 2E and F).

### In vitro study reveals HOXC6 is upregulated in HNSCC cell lines

To ascertain that HOXC6 is upregulated in HNSCC, we validated its expression at the RNA level using qRT-PCR in HNSCC cancer cell lines (UMSCC-047, UPCI-SCC-090, UMSCC-104, and 93VU-147T) compared to that in non-cancerous cell lines (OKF6 and HOK16B), which served as controls. Our analysis revealed that compared to the controls, HOXC6 expression was significantly increased in the HNSCC cell lines, specifically in the 93VU-147T and UMSCC-047 cell lines (Supplementary Fig. 3). Our TCGA data were consistent with our in vitro analysis. As the UMSCC-047 and 93VU-147T cell lines displayed the highest expression of HOXC6 (Supplementary Fig. 3), they were selected for further studies.

### Downregulation of HOXC6 reduces cell proliferation and migration in HNSCC cell lines

**Fig. 2:**
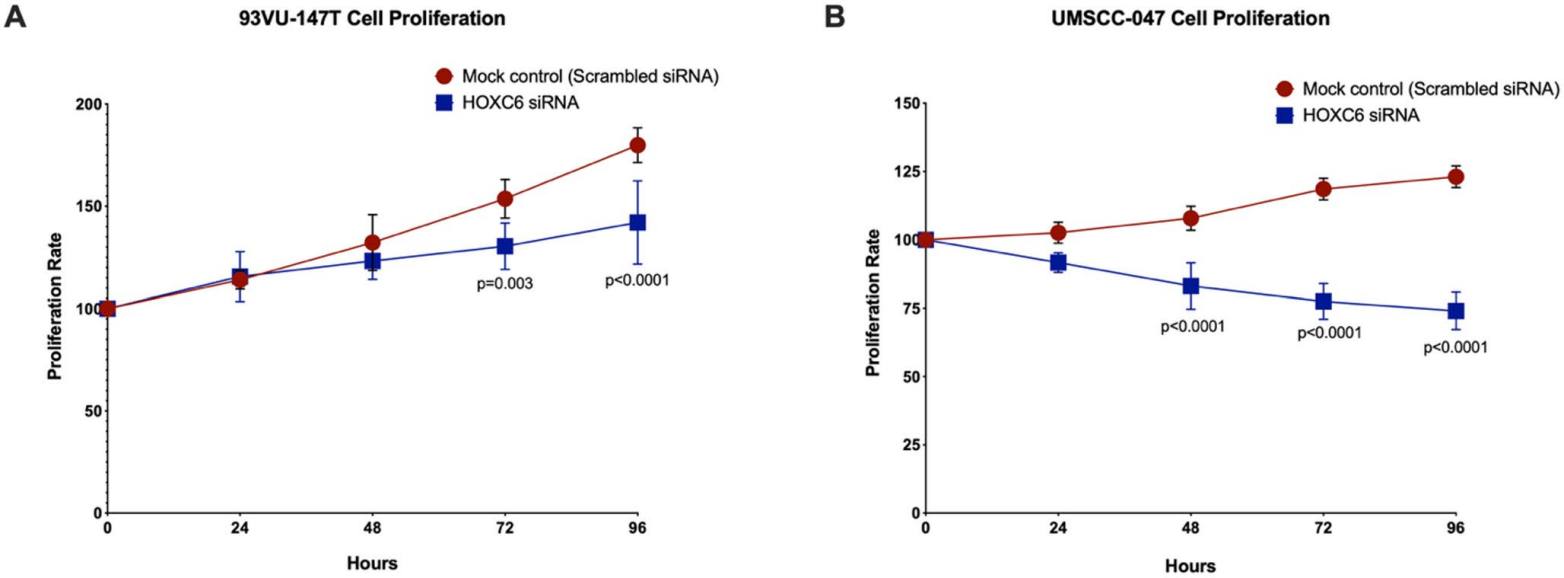
Effect of HOXC6 on the cell proliferation rate in HNSCC cell lines. The Alamar Blue cell proliferation assay was performed on two HNSCC cell lines, (**A**) 93VU-147T and (**B**) UMSCC-047, across different time intervals (0, 24, 48, 72, and 96 h) following siRNA-mediated HOXC6 knockdown or non-targeting (scramble control) siRNA. Fluorescence intensity was measured at each time point, and the relative growth curve was plotted for the control (red) and the HOXC6 siRNA (blue). The figure shows that depletion of HOXC6 decreases the cell proliferation rate after 72 h for the 93VU-147T cell line and after 48 h for the UMSCC-047 cell line, compared to the control. The scale is indicated on the left side (O.D./Time points), and the error bars represent the standard deviation

**Fig. 3:**
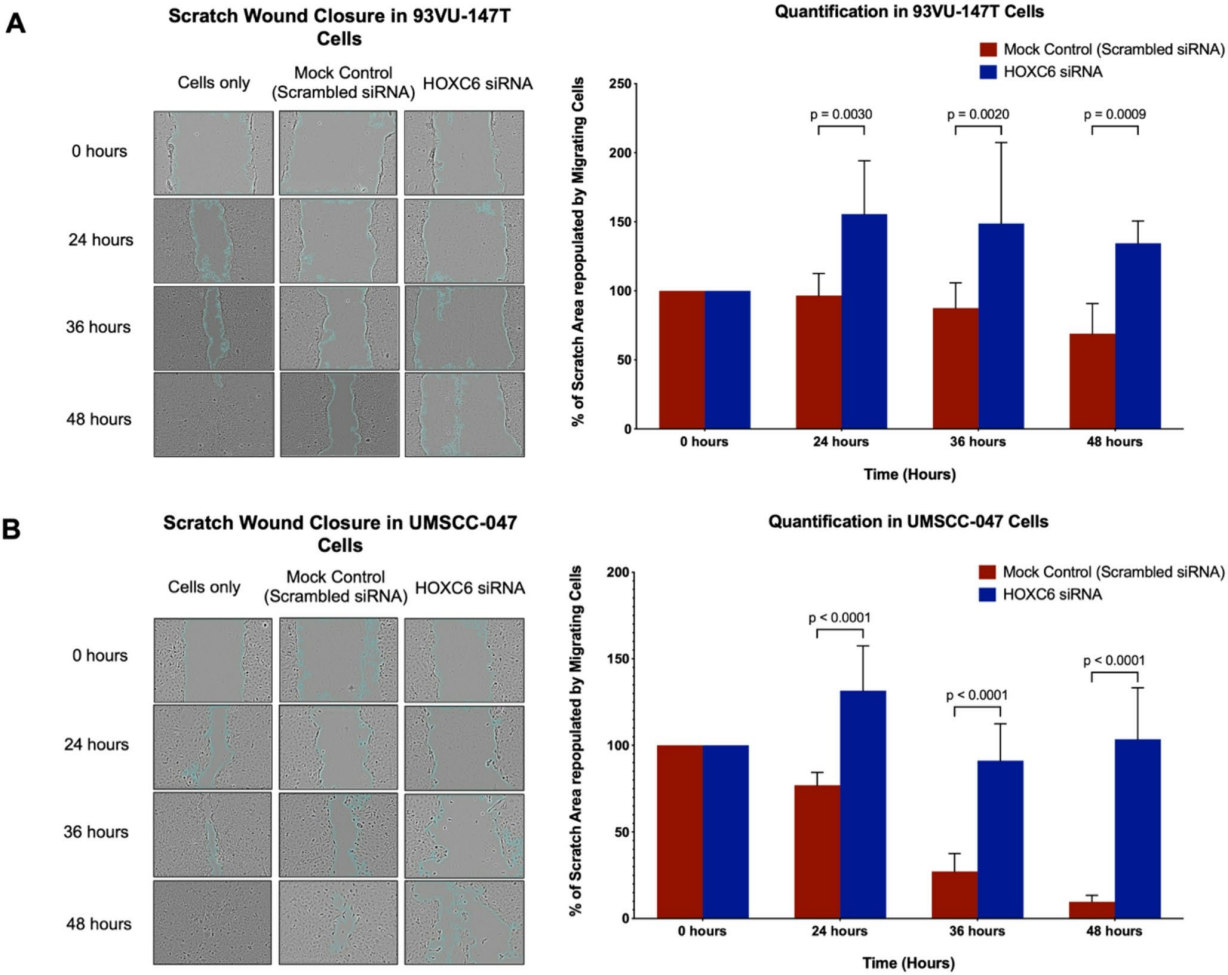
(A and B). Effect of HOXC6 on wound healing closure in HNSCC cell lines. Representative images from in vitro scratch wound healing assays in (**A**) 93VU-147T and (**B**) UMSCC-047 cells at different time intervals (0, 24, 36, and 48 h) upon siRNA-mediated HOXC6 knockdown or the non-targeting siRNA treatment. The wound healing assay was quantified using the Fiji (ImageJ) software, and the migration rates were quantified by measuring three different wound areas. Bar graphs were plotted and represent mean-SD values of one representative experiment. Statistical significance was obtained when *p* < 0.05

To determine the oncogenic potential of HOXC6 in HNSCC, we investigated the effect of siRNA-mediated HOXC6 knockdown on the physiology of HNSCC cells (Figs. 2 and 3).

Following successful downregulation in both cell lines, we analyzed the proliferative ability of the transfected cell lines in comparison with their scrambled control using the Alamar blue cell proliferation assay, which revealed that depletion of HOXC6 (Supplementary Fig. 4) resulted in a significant decrease in cell proliferation (Fig. 2A and B). Specifically, in the UMSCC-047 cell line, we observed a decrease in the proliferation rate between the control (red) and HOXC6 siRNA (blue) at 48 h. Conversely, in the 93VU-147T cell line, this decrease in proliferation became more visible at 72 h, with a further reduction throughout the time course. This indicated that HOXC6 had a significant effect on cell proliferation, specifically in the UMSCC-047 cell line. In summary, these results demonstrated that HOXC6 expression is associated with increased cell proliferation in HNSCC.

### HOXC6 silencing influences genes involved in immune response and cell cycle pathways

To elucidate the molecular mechanism of HOXC6, we silenced the expression of HOXC6 in 93VU-SCC-147T and UMSCC-047 cells using an siRNA specific to HOXC6 or a non-targeting siRNA. We extracted RNA from each cell line in triplicate and performed RNA sequencing, as described in the Materials and Methods section.

**Fig. 4:**
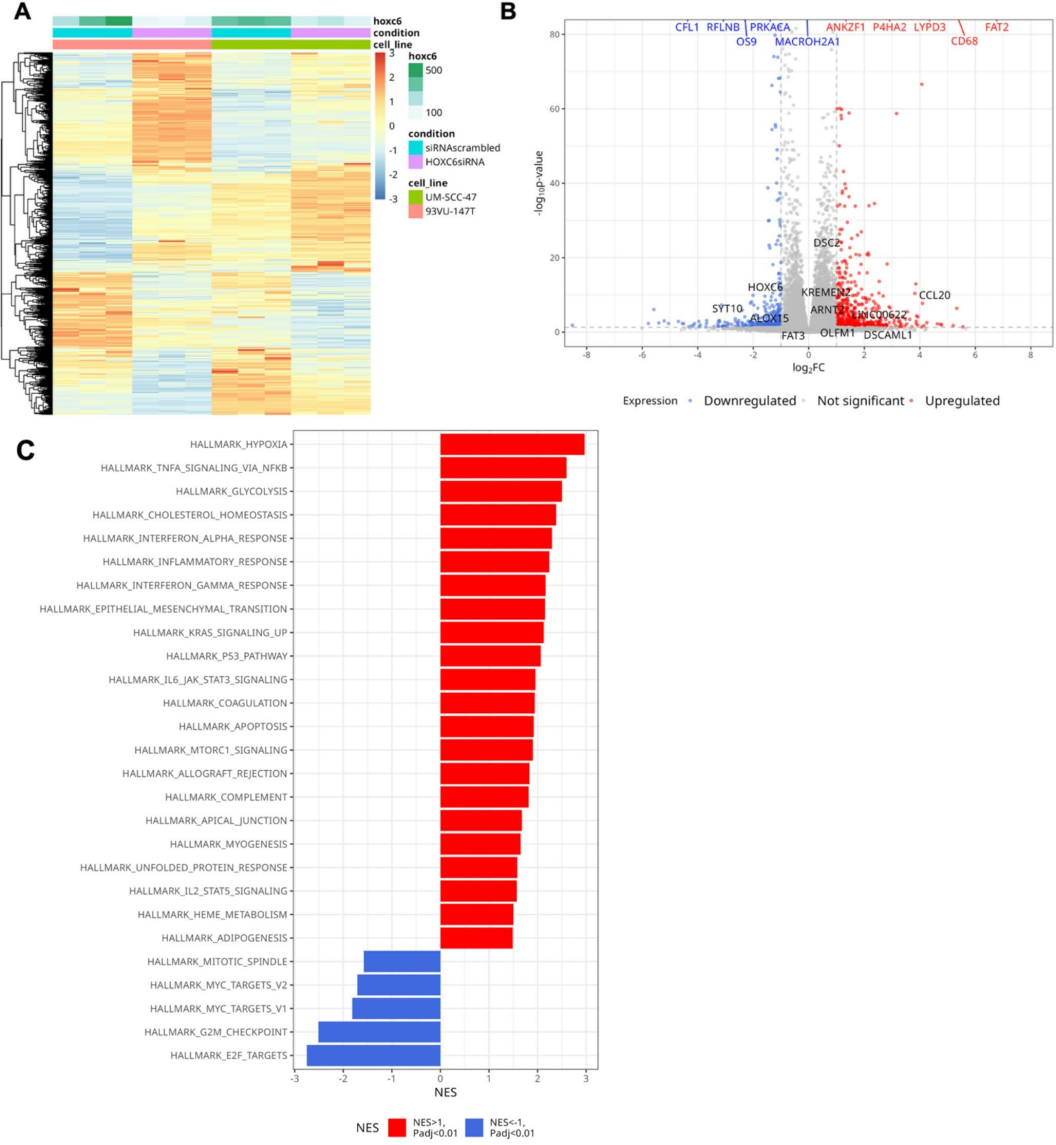
(**A-C**). Transcriptomic Alterations and Pathway Enrichment in HNSCC cell lines following HOXC6 knockdown (**A**). Differential gene expression heatmap comparing HOXC6 siRNA knockdown versus scrambled siRNA control in two cell lines, UMSCC-47 and 93VU-147T. This heatmap was built using DESeq2 on normalized gene read counts. Rows represent individual genes, while columns represent experimental conditions. Data is filtered for genes with an adjusted p-value < 0.05 and an absolute log2 fold change > 1. The heatmap displays normalized counts scaled by row, with red indicating upregulation and blue indicating downregulation. Annotations above the heatmap denote the HOXC6 expression level, experimental condition (scrambled siRNA or HOXC6 siRNA knockdown), and cell line. Hierarchical clustering shows distinct gene expression patterns in response to HOXC6 knockdown. (**B**). A Volcano plot depicting differential gene expression in scrambled siRNA control versus HOXC6 siRNA knockdown. The x-axis represents log2 fold change (LFC) of siRNA-HOXC6 vs. siRNA control, and the y-axis represents the -log10 p-value with a maximum cutoff on -log10 p-value = 85. Genes significantly upregulated (red) and downregulated (blue) are highlighted, with nonsignificant genes shown in gray. The top differentially expressed genes are labeled (red and blue colors according to the LFC. The dashed lines represent the significance threshold (adjusted p-value < 0.05, |LFC|>1). (**C**). Bar plot showing hallmark gene sets enriched in HOXC6 siRNA knockdown samples based on Gene Set Enrichment Analysis (GSEA). The x-axis represents the normalized enrichment score (NES), with red bars indicating upregulated pathways (NES > 1, adjusted p-value < 0.01) and blue bars indicating downregulated pathways (NES <−1, adjusted p-value < 0.01) upon HOXC6 silencing. Pathways are ranked by NES, highlighting the most significantly enriched processes. (**D**). A Volcano plot from Fig. 4C depicting differential gene expression in scrambled siRNA control versus HOXC6 siRNA knockdown, with key EMT and stemness genes labelled

We identified 889 genes that significantly changed in expression following HOXC6 silencing (padj < 0.05, absolute log2 fold change > 1) (Fig. 4A and B). Detailed statistical information for all the identified genes is provided in Supplementary Table S3A. Among these, 350 genes were significantly downregulated, whereas 539 genes were upregulated after HOXC6 silencing, highlighting the dual regulatory role of HOXC6 in gene silencing and activation. Some of the most downregulated genes represented in the volcano plot include OS9 (ER homeostasis and suppression of cellular stress responses), MACROH2A1 (which replaces conventional H2A histones in specific nucleosomes to repress transcription), CFL1 (which regulates actin polymerization), RFLNB (a filamin-binding protein), and PRKACA (a catalytic subunit of protein kinase A), suggesting that their expression is directly regulated by or correlated with HOXC6. Therefore, HOXC6 silencing downregulates multiple genes involved in chromatin remodeling, cytoskeletal organization, and signal transduction, indicating that HOXC6 may function as a transcriptional activator that promotes oncogenic pathways. Conversely, the top upregulated genes included tumor suppressor FAT2, CD68 (macrophage marker, immune response), LYPD3 (which is predicted to mediate laminin-binding activity), P4HA2 (involved in collagen synthesis), and ANKZF1 (a tRNA endonuclease). This observation demonstrates that HOXC6 may repress genes involved in tumor suppression, immune signaling, ECM remodeling, and RNA regulation, reinforcing its oncogenic potential. This highlights HOXC6’s dual role in maintaining an oncogenic state by repressing tumor suppressors and activating pro-tumorigenic pathways.

GSEA revealed significant positive enrichment of several MSigDB cancer hallmark gene sets after HOXC6 silencing (Normalized Enrichment Score (NES) > 1, padj < 0.01), indicating a negative correlation with HOXC6 expression (Supplementary Table S3B). These include multiple immune response-related hallmarks, TNF signaling via NF-κB, interferon-alpha response, inflammatory response, interferon-gamma response, p53 signaling, hypoxia, cholesterol homeostasis, and glycolysis (Fig. 4C). These results suggested that HOXC6 may act as a negative regulator of these pathways. Notably, hallmark gene sets associated with the G2/M checkpoint and E2F targets were negatively enriched after HOXC6 silencing (NES <−1, padj < 0.01), indicating that HOXC6 expression positively correlates with the activity of the G2/M checkpoint and E2F targets. These observations further suggested that the activity of these hallmark gene sets is regulated by HOXC6 expression. Since both the G2/M and E2F hallmark gene sets are typically active in most cancers, their strong association with high HOXC6 expression indicates their potential involvement in regulating cell cycle progression (Fig. 4C). However, HOXC6 silencing led to significant enrichment of the epithelial-mesenchymal transition (EMT) pathway (Fig. 4C). We further performed functional validation of the pathway enrichment data using RNA-sequencing-based expression analysis of canonical EMT and stemness genes in the HNSCC cell lines (UMSCC-047 and 93VU-147T) (Supplementary Fig. 5). HOXC6 silencing resulted in a transcriptional shift characterized by reduced EMT marker expression and increased mesenchymal and stemness-associated genes, indicating activation of EMT and acquisition of stemness traits (Supplementary Fig. 5). Specifically, while E-cadherin expression was reduced, expression of vimentin, fibronectin, N-cadherin, P-cadherin, b-catenin, c-Myc and CD44 were upregulated following HOXC6 depletion (Supplementary Fig. 5). Expression of SOX2 and SALL4 remained largely unchanged, whereas OCT4 showed variable induction (Supplementary Fig. 5), suggesting partial regulation of stemness networks. This suggests that HOXC6 may have a more complex and context-dependent role in regulating cellular plasticity.

**Fig. 5:**
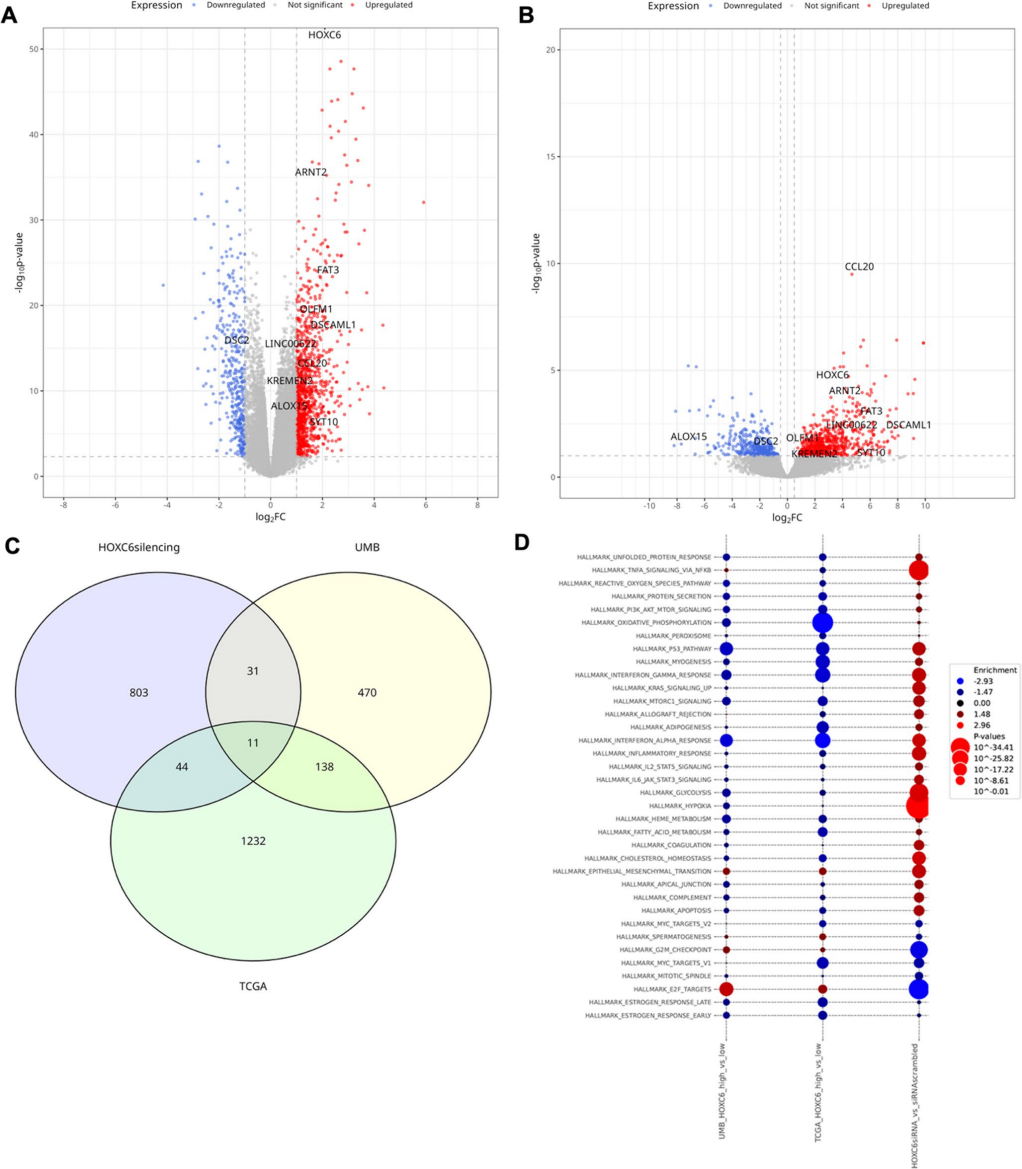
Differential gene expression in the TCGA and UMB HNSC cohorts under high and low HOXC6 levels conditions. (**A**). Volcano plot demonstrates differentially expressed genes in the TCGA HNSCC cohort stratified by HOXC6 expression. Tumor samples were classified into low and high HOXC6 expression based on the median expression value. Upregulated genes (LFC > 1, padj < 0.05 threshold) are marked in red, and downregulated genes (LFC<−1, padj < 0.05 threshold) are marked in blue. (**B**). Volcano plot demonstrates differentially expressed genes after comparison of HNSC UMB samples with the top 5 HOXC6 expression vs. samples with the bottom 5 HOXC6 expression. Upregulated genes (LFC > 1, padj < 0.05 threshold) are marked in red, and downregulated genes (LFC<−1, padj < 0.05 threshold) are marked in blue. (**C**). The Venn diagram highlights the overlap of differentially expressed genes between the HNSCC cell lines (93VU-147T and UMSCC-047) after HOXC6 knockdown, TCGA and UMB datasets, showing common and unique gene sets across the comparisons. (**D**) GSEA was conducted to identify hallmark cancer gene sets with expression correlating with HOXC6 expression in the TCGA cohort, UMB cohort, as well as in the HNSCC cell lines (93VU-147T and UMSCC-047) after HOXC6 knockdown. Each dot represents a gene set enrichment in a specific experiment; only sets with p-value < 0.01 are shown. The color of the dot indicates normalized enrichment score (NES), while the size of the dot corresponds to the adjusted p-values, so that larger-sized dots represent gene sets with significant differential expression between samples with high and low HOXC6 expression levels

We further evaluated the concordance of HOXC6-related data in the cell line models and clinical samples. We first performed differential expression analysis of HNSCC tumor samples from the TCGA cohort by stratifying samples into HOXC6-high and HOXC6-low expression groups, using the median HOXC6 mRNA expression level as the cutoff (Fig. 1E). The analysis identified 24,242 genes in the read count matrix (Supplementary Table S4A), of which only 1,425 differentially expressed genes (DEGs) were identified as differentially expressed (padj < 0.05 and |LFC| > 1), including both upregulated (LFC > 1) and downregulated genes (LFC<−1). To visualize transcriptional differences, a volcano plot was generated (Fig. 5A).

Additionally, a similar analytical approach was applied to the HNSCC UMB cohort, comparing five tumor samples with the highest and five with the lowest HOXC6 median expression levels (Fig. 5B). Despite the small size of the UMB cohort and its different clinical profiles, we identified 650 DEGs between the HOXC6-high and HOXC6-low samples (Supplementary Table S4B). Notably, 149 genes were differentially expressed in both TCGA and UMB HNSCC cohorts (Fig. 5B and C). To determine whether HOXC6 directly regulates genes with correlated expression, we intersected DEGs from TCGA and UMB clinical samples with DEGs identified in the HOXC6 silencing experiments. Among the TCGA DEGs, 55 genes exhibited altered expression following HOXC6 silencing, of which 11 genes, including HOXC6, were differentially expressed in the UMB cohort (Fig. 5C) (Supplementary Table S4C). This overlap underscores their potential relevance to HOXC6-mediated transcriptional regulation and highlights a subset of genes whose expression is not only consistently associated with HOXC6 levels in clinical HNSCC tumor samples but also potentially regulated by HOXC6.

To summarize the effect of HOXC6 expression on other genes, both in vitro and in patient samples, we performed GSEA on DEGs from TCGA HOXC6-high vs. HOXC6-low (Supplementary Table S5A), UMB HOXC6-high vs. HOXC6-low (Supplementary Table S5B), and HOXC6 knockdown versus scramble control datasets (Supplementary Table S5C), focusing on MSigDB hallmark cancer gene sets (Fig. 5D). In our earlier observations, HOXC6 knockdown led to significant positive enrichment of immune-response hallmark pathways, indicating an inverse correlation between HOXC6 expression and immune activation. In contrast, analysis of TCGA and UMB datasets showed negative enrichment of these same immune pathways in tumors with high HOXC6 expression, supporting the observation that HOXC6 may suppress immune-related gene programs. Similarly, proliferative pathways such as E2F targets and the G2M checkpoint were positively enriched in HOXC6-high tumors in both datasets but showed negative enrichment following HOXC6 silencing, suggesting that HOXC6 promotes cell cycle-related transcriptional programs. These findings indicated that HOXC6 expression intricately influences immune signature genes and cell cycle-related pathways.

### HOXC6 overexpression in HNSCC is associated with H3K27ac-mediated promoter acetylation

**Fig. 6:**
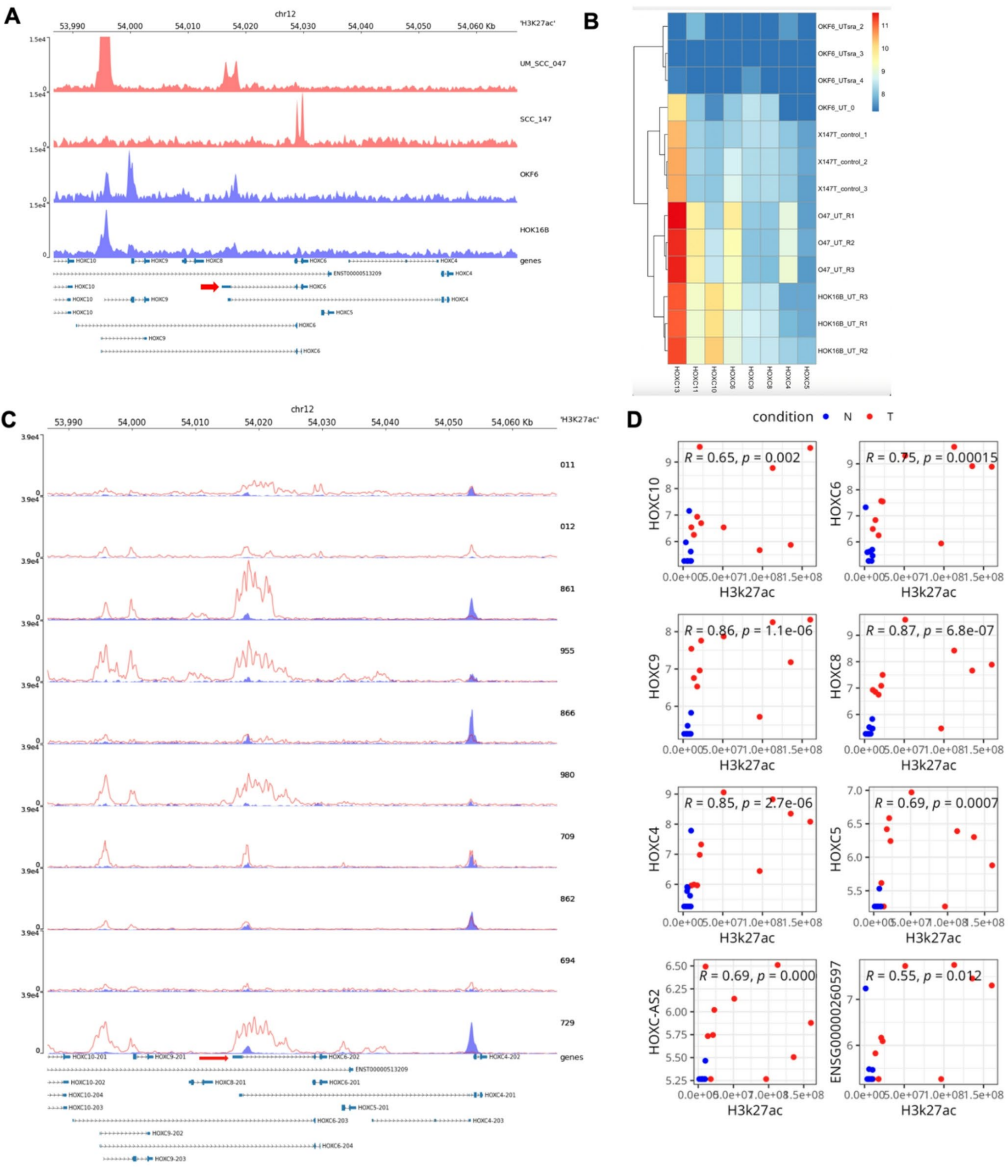
High HOXC6 expression is driven by histone acetylation at the promoter region in HNSCC.(**A**) IGV view of ChIP-seq profiles of H3K27ac histone modifications at the HOXC locus (chromosome 12: 53,990 − 54,060 Kb) in 93VU-147T and UMSCC-047 cells (red tracks), representing HNSCC cell lines, and in OKF6 and HOK16B (blue tracks), representing non-cancerous cell lines. The y-axis indicates the read density, reflecting H3K27ac enrichment at specific genomic regions. Peaks denote active regulatory regions, highlighting differences in enhancer activity between cancerous and non-cancerous cells. Annotated genes within the HOXC locus are shown below, indicating the genomic position of HOXC family members and associated transcripts. The red arrow indicates the HOXC6 gene locus. (**B**). Heatmap showing the expression levels of HOXC genes (HOXC10, HOXC9, HOXC8, HOXC6, and HOXC5) across different cell lines, including cancerous (93VU-147T and UMSCC-047) and non-cancerous (OKF6 and HOK16B) cells. Rows represent samples, and columns represent genes. Expression levels are indicated by a gradient color scale, with red representing higher expression and blue representing lower expression (log-transformed values). Hierarchical clustering of both samples and genes highlights distinct patterns of gene expression, differentiating cancerous and non-cancerous cell lines. (**C**). Chromatin landscape at the HOXC locus on chromosome 12, spanning positions 53,990 kb to 54,060 kb, depicting H3K27ac enrichment in UMB patient samples (10 paired tumor and normal samples). The red tracks represent the H3K27ac ChIP-seq signal in the 10 tumor samples, while the blue tracks correspond to input controls or baseline signals. Peaks indicate regions of active chromatin, suggesting transcriptional regulatory activity. Gene annotations for HOXC10, HOXC9, HOXC8, HOXC6, HOXC5, and adjacent loci are shown below, providing context for the genomic regions analyzed. Variations in H3K27ac levels across samples highlight differential enhancer activity associated with the HOXC cluster. The red arrow indicates the HOXC6 gene locus (**D**). Correlation between H3K27ac enrichment and gene expression for HOXC cluster genes across normal (N) and tumor (T) samples. Each scatter plot represents this relationship for a specific gene (HOXC10, HOXC6, HOXC9, HOXC8, HOXC4, and HOXC5) together with the Spearman correlation coefficient and the corresponding p-value indicated in the top left of each panel. Blue and red dots correspond to normal (N) and tumor (T) samples, respectively

Next, we investigated the regulation of HOXC6 expression. To explore this, we performed ChIP-seq using H3K27ac, a histone modification in the active enhancer and promoter regions of genes. ChIP was conducted in HNSCC cell lines (UMSCC-047 and 93VU-147T) and control cell lines (OKF6 and HOK16B), followed by high-throughput sequencing. ChIP-seq results showed higher H3K27ac enrichment (red tracks) near the HOXC6 promoter in HNSCC cell lines, specifically in UMSCC-047 cell lines, compared to the control cell lines (Fig. 6A). We paired ChIP-seq data with the expression profiles of the HOXC gene cluster (including HOXC6) obtained from RNA-seq of the same cell line (Fig. 6B) (Supplementary Fig. 6). As expected, HOXC6 expression was comparable in UMSCC-047 cell lines with H3K27ac enrichment, in contrast to other control cell lines. The heatmap also revealed a strong correlation between the expression of several other HOXC family genes, particularly HOXC4 and HOXC11, in the HNSCC cell lines.

We extended our analysis to the UMB HNSCC primary cohort samples. A similar ChIP-seq study was performed using the H3K27ac mark. Higher H3K27ac enrichment was observed at the HOXC6 promoter region (red arrow) in most of the tumor samples (red tracks) relative to normal controls (blue tracks) (Fig. 6C). Interestingly, we also observed differential histone acetylation activity within the HOXC gene cluster in the tumor samples. Sample-wise comparison between H3K27ac ChIP-seq and RNA-seq HOXC gene expression profiles demonstrated a good correlation between the size of the H3K27ac peak and the expression intensity for HOXC6 and most of the other HOXC genes. Consistent with TCGA data, we observed elevated levels of HOXC6 expression in most of the primary tumor samples compared to the standard control samples (Fig. 6D). These findings suggest that H3K27ac-mediated acetylation of the HOXC6 promoter may drive its upregulation in HNSCC, potentially influencing cell differentiation, behavioral regulation, and tumor progression.

To determine whether HOXC6 overexpression is regulated by the BET protein BRD4, we examined the effect of the BET inhibitor JQ1 on HOXC6 expression. BET proteins, such as BRD4, bind to acetylated histones (e.g., H3K27ac) at active promoters and enhancers to activate transcription, a process disrupted by JQ1 through competitive inhibition of BRD4 binding [42]. We analyzed the RNA sequencing data from UMSCC-047 cells treated with JQ1 and DMSO-treated controls. Our preliminary analysis revealed that JQ1 treatment reduced HOXC6 expression compared to that in DMSO-treated control samples (Supplementary Fig. 7 A). To validate this interaction, we performed ChIP-qPCR for BRD4 at the HOXC6 promoter in normal oral keratinocytes, OKF6, and HNSCC cells, UMSCC-047. BRD4 enrichment at the HOXC6 promoter was markedly higher in UMSCC-047 cells compared to OKF6, confirming direct BRD4 binding to the promoter region (Supplementary Fig. 7B). Together, these findings provide mechanistic evidence linking H3K27ac-mediated promoter acetylation and BRD4 occupancy to HOXC6 overexpression, suggesting that JQ1 may serve as a potential therapeutic agent for targeting HOXC6 in HNSCC. 

## Discussion

Although aberrant HOXC6 expression has been reported in various cancer types [14–18], the mechanisms underlying the function and regulation of this gene in HNSCC cells remain unclear. In this study, we explored the role of HOXC6 in HNSCC. Analysis of TCGA datasets revealed that HOXC6 is significantly overexpressed in HNSCC, and its increased expression of HOXC6 is associated with poor survival. Notably, high HOXC6 expression was correlated with lymph node metastasis (N stage), suggesting a potential role in promoting tumor spread.

Our in vitro analyses further confirmed the upregulation of HOXC6 expression in HNSCC cell lines. Functional assays following HOXC6 knockdown demonstrated a reduced cell proliferation rate and migration, indicating the oncogenic potential of HOXC6. These findings align with those of previous studies in human cancers, including esophageal squamous cell carcinoma, where HOXC6 promotes migration, invasion, and proliferation of cells by modulating the expression of genes involved in malignant phenotypes [12, 16, 43, 44].

Our study revealed a novel and multifaceted role for HOXC6 in HNSCC, particularly in modulating immune responses and key oncogenic pathways. Silencing of HOXC6 expression led to significant transcriptional changes, uncovering its role in promoting cell cycle progression and immune modulation. Specifically, HOXC6 positively regulated genes associated with the G2/M checkpoint and E2F target pathways, both of which are crucial for cell cycle control. The E2F family of transcription factors drives the transition from G1 to S phase, promoting DNA synthesis and cell cycle progression [45], while G2/M checkpoint genes ensure proper mitotic entry and chromosome segregation [46]. Dysregulation of these pathways is often observed in HOXC6-overexpressing tumors, leading to uncontrolled cell cycle progression, increased cell proliferation, and genomic instability, which are the hallmarks of cancer progression [47]. These findings are consistent with previous findings in prostate cancer, where HOXC6 silencing also reduced the E2F and G2/M checkpoint-related pathways, reinforcing its pro-proliferative role [48, 49].

Furthermore, GSEA analysis from TCGA and primary sample data (UMB) revealed that immune response-related pathways were significantly downregulated in tumors with high HOXC6 expression. This implies that HOXC6 may contribute to immune evasion by suppressing immune-activating genes and fostering an immunosuppressive tumor microenvironment. Potential mechanisms may include the recruitment of immunosuppressive cell types such as regulatory T cells (Tregs) and myeloid-derived suppressor cells (MDSCs). Interestingly, this immune suppression may paradoxically stimulate immune cell infiltration in response to increased tumor antigen release, possibly driven by enhanced proliferation mediated by HOXC6-regulated E2F and G2/M genes. Together, these findings suggest that HOXC6 is a central regulator of HNSCC tumor biology, with dual roles in promoting cell dysregulation and shaping an immunosuppressive microenvironment. Additionally, enrichment of the H3K27ac mark at the promoter regions of HOXC6 in both cancer cell lines and primary samples suggests that the HOXC6 promoter is epigenetically active and driven by epigenetic mechanisms such as histone acetylation.

Our study had several limitations. First, the majority of the experiments were performed using in vitro models that do not fully mimic the biological complexity of the in vivo tumor microenvironment. Therefore, prospective validation of the oncogenic potential of HOXC6 in animal models is necessary to substantiate our findings and evaluate the potential of HOXC6 as a therapeutic target. Second, the small number of UMB patient samples limited the generalizability of our observations regarding the broader role of HOXC6 in HNSCC. Moreover, tumor heterogeneity in HNSCC complicates the interpretation of our results.

**Fig. 7:**
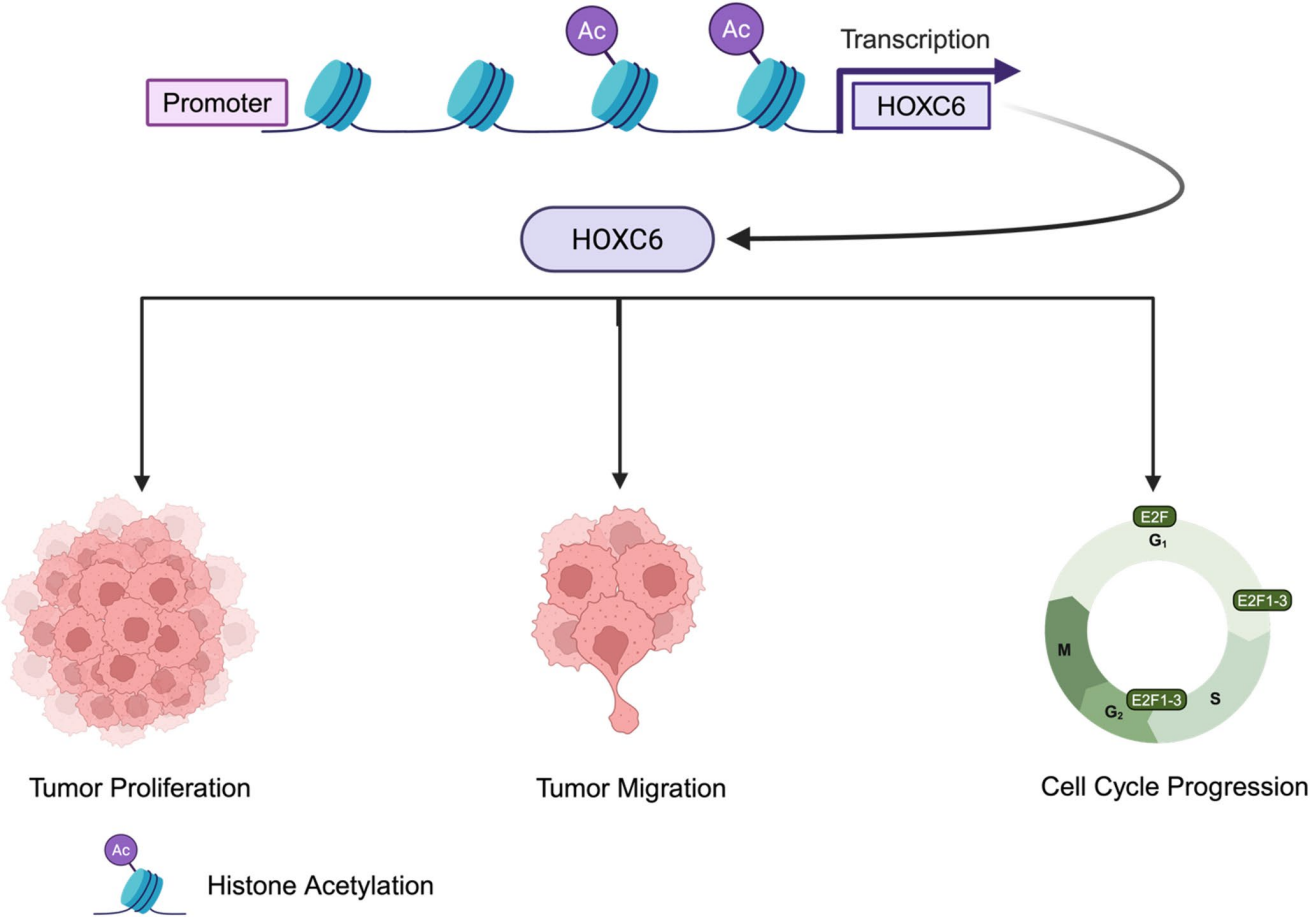
**Mechanistic model depicting HOXC6 overexpression and its oncogenic effects in HNSCC**.H3K27ac CHIP-seq analysis reveals histone acetylation at the promoter region of HOXC6, enhancing transcription and leading to overexpression of HOXC6 in HNSCC. Elevated HOXC6 levels contribute to HNSCC progression by promoting cell proliferation, migration, and cell cycle progression. RNA-seq and GSEA indicate that HOXC6 overexpression is associated with dysregulation of the cell cycle, particularly through the activation of E2F transcription factors and G2/M checkpoint transitions, facilitating uncontrolled cell division and tumor growth. Figure created in https://BioRender.com

In conclusion, our study demonstrated that HOXC6 overexpression in HNSCC is linked to poor overall survival and enhanced tumor aggressiveness through the modulation of cell cycle (Fig. 7). These findings suggest that HOXC6 is a promising prognostic biomarker and potential therapeutic target. However, further studies are necessary to evaluate the significance of HOXC6 as a predictive and prognostic factor before establishing its clinical utility.

## Supplementary Information

Below is the link to the electronic supplementary material.

Supplementary Material 1 (XLSX 8.15 KB)

Supplementary Material 2 (XLSX 193 KB)

Supplementary Material 3 (XLSX 2.10 MB)

Supplementary Material 4 (XLSX 4.85 MB)

Supplementary Material 5 (XLSX 39.0 KB)

Supplementary Material 6 (XLSX 9.61 KB)

Supplementary Material 7 (PDF 887 KB)

## Data Availability

All data supporting the findings of this study are available in the paper and its Supplementary Information section. The raw RNA-seq data have been submitted to the dbGaP and GEO databases and will be publicly available upon completion of the deposition process. Accession numbers will be provided once available.
