# Supplementary material for: HOXC6 overexpression stimulates cell migration and correlates with poor prognosis in head and neck squamous cell carcinoma
Source: Cell Mol Life Sci. 2026 Jan 11;83(1):77. doi: 10.1007/s00018-025-06039-3 (PMC12858702; doi:10.1007/s00018-025-06039-3)
Supplement: Supplementary file 7 — Supplementary Material 7 (PDF 887 KB) [file 18_2025_6039_MOESM7_ESM.pdf]

Supplementary Figures

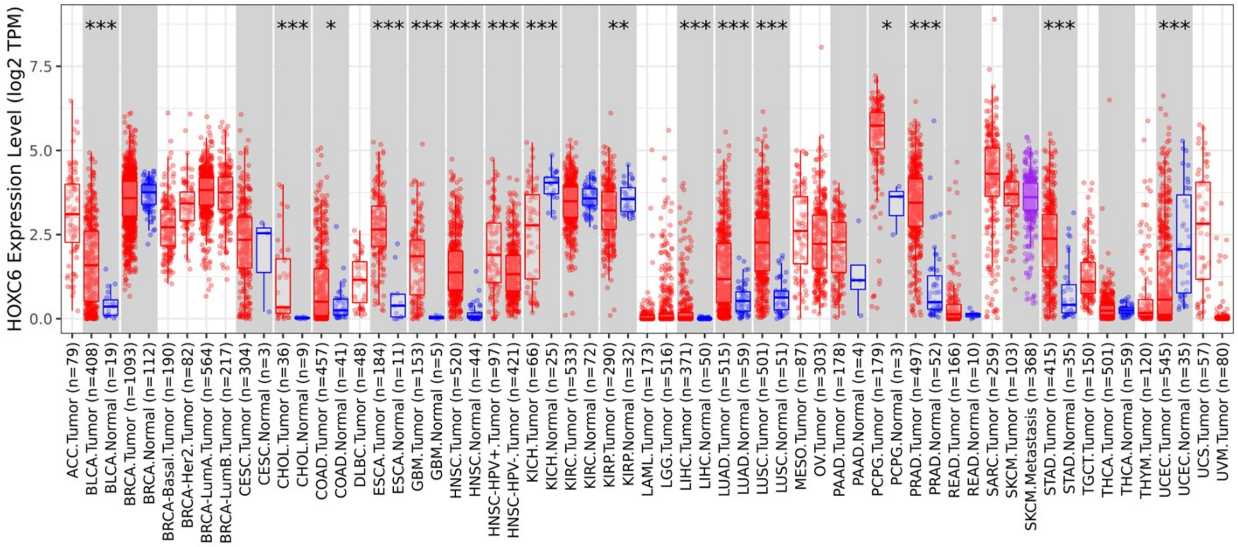

Supplementary Figure 1. Pan-Cancer analysis was performed for the expression of HOXC6 using the Timer2.0 software. The box plots show a significant upregulation of HOXC6 expression in several tumors as compared to their regular counterparts. Notably, HOXC6 was significantly upregulated in HNSCC and was selected for further studies as an oncogenic candidate in HNSCC.

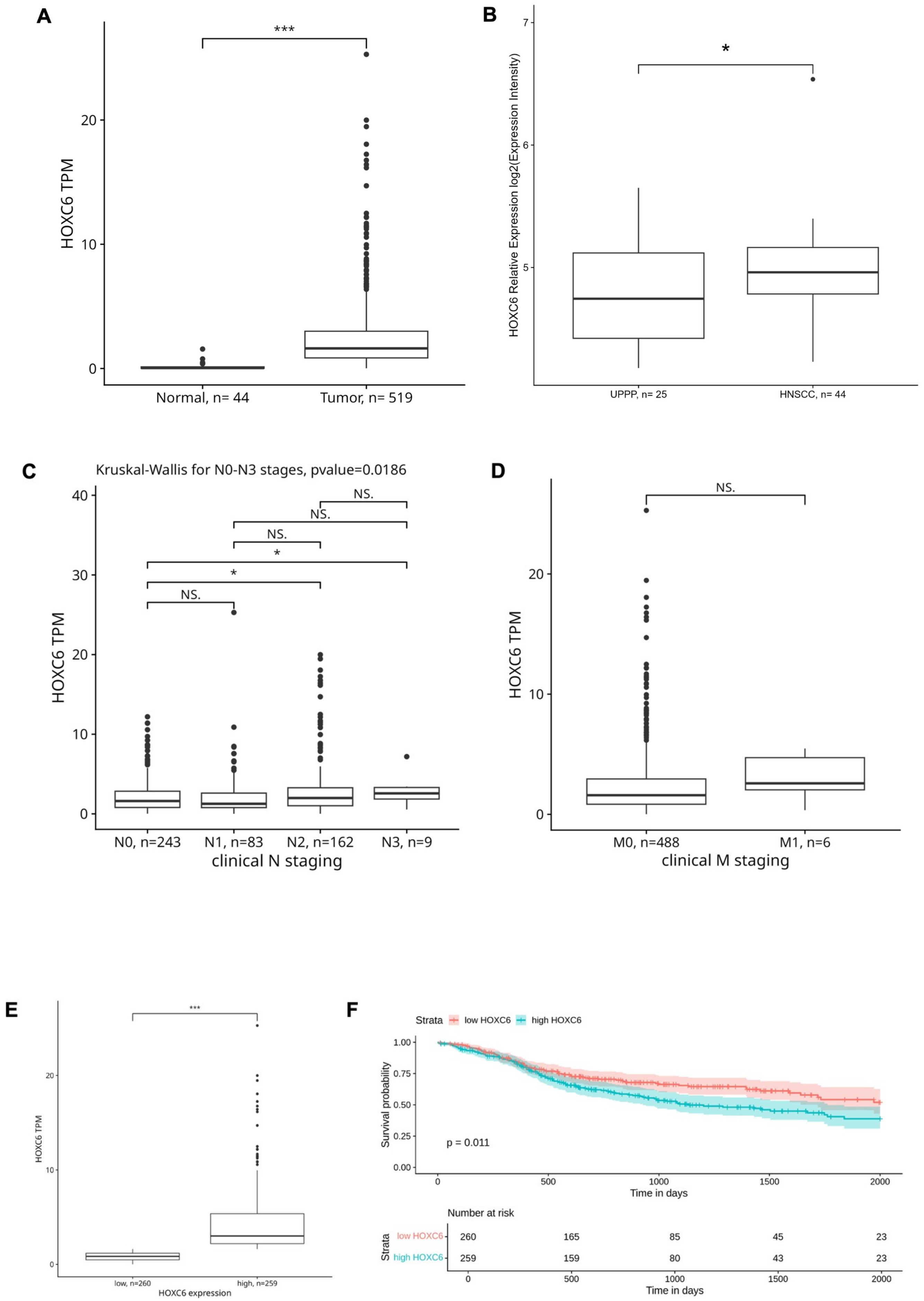
A
***
HOXC6 TPM
20
10
0
Normal, n= 44
Tumor, n= 519
B
*
HOXC6 Relative Expression log2(Expression Intensity)
7
6
5
UPPP, n= 25
HNSCC, n= 44
C
Kruskal-Wallis for N0-N3 stages, pvalue=0.0186
NS.
NS.
NS.
*
*
NS.
40
30
20
10
0
HOXC6 TPM
N0, n=243
N1, n=83
N2, n=162
N3, n=9
clinical N staging
D
NS.
HOXC6 TPM
20
10
0
M0, n=488
M1, n=6
clinical M staging
E
***
HOXC6 TPM
20
10
0
low, n=260
high, n=259
HOXC6 expression
F
Strata
low HOXC6
high HOXC6
1.00
0.75
0.50
0.25
0.00
Survival probability
p = 0.011
0
500
1000
1500
2000
Time in days
Number at risk
Strata
low HOXC6 260 165 85 45 23
high HOXC6 259 159 80 43 23
0
500
1000
1500
2000
Time in days

Supplementary Figure 2 (A-F). (A). TCGA analysis revealed that HOXC6 expression was significantly ($p < 0.0001$) upregulated in HNSCC tissues (mean = 2.115 TPM) (n=519) in comparison to normal tissue samples (mean = 0.05250 TPM) (n=44). **(B).** Analysis of a gene expression microarray cohort (GSE33205) reveals significantly ($p = 0.035$, Welch's t-test) higher expression in HNSCC tissues compared to uvulopalatopharyngoplasty (UPPP) controls. **(C).** Differences between the medians among different N stages were evaluated using the Kruskal-Wallis test, followed by the Dunn test with Benjamini-Hochberg corrections for multiple testing. The median HOXC6 expression significantly differs among N stages (Kruskal-Wallis, p=0.018), specifically between N0 and N2-N3 stages (Dunn, padj < 0.05). **(D)** TCGA analysis of clinical M stage. HOXC6 expression in different M cancer stages. Differences between the medians among different M stages were evaluated using the Wilcoxon rank sum test. Due to the small TCGA cohort size of M1 stage (n=6), we did not obtain a positive correlation between HOXC6 expression and M stage. **(E) Classification of tumors based on the median expression of HOXC6.** For the survival analysis, tumors were classified based on the median expression of HOXC6 (2.109 TPM) into low and high HOXC6 gene expression levels. The classification was statistically significant according to the Mann-Whitney Test ($p < 0.001$). **(F).** Kaplan-Meier survival curves for the association of HOXC6 expression with overall survival across HNSCC. High and low expression levels were defined using the median transcriptomic expression of HOXC6 (2.109 TPM). The x-axis denotes time in days, and the y-axis represents the proportion of patients surviving. Statistical significance is determined using log-rank tests. The divergence or convergence of the curves indicates the potential impact of HOXC6 expression on overall survival outcomes in HNSCC. Patients with higher (2.109 TPM and above) HOXC6 expression in tumor samples have worse survival rates compared to the patients with lower (below 2.109 TPM) HOXC6 expression (p=0.01).

Supplementary Figure 3. HOXC6 expression in non-cancerous and HNSCC cell lines. HOXC6 expression was evaluated in the non-cancerous cell lines (red), HOK16B and OKF6, in comparison to the HNSCC cell lines (blue), UPCI-SCC-090, UMSCC-104, 93VU-147T, and UMSCC-047. The y-axis indicates relative HOXC6 expression from qRT-PCR data. The error bar represents the standard deviation. Exact p-values are indicated on the graph. $p < 0.05$ was considered significant.

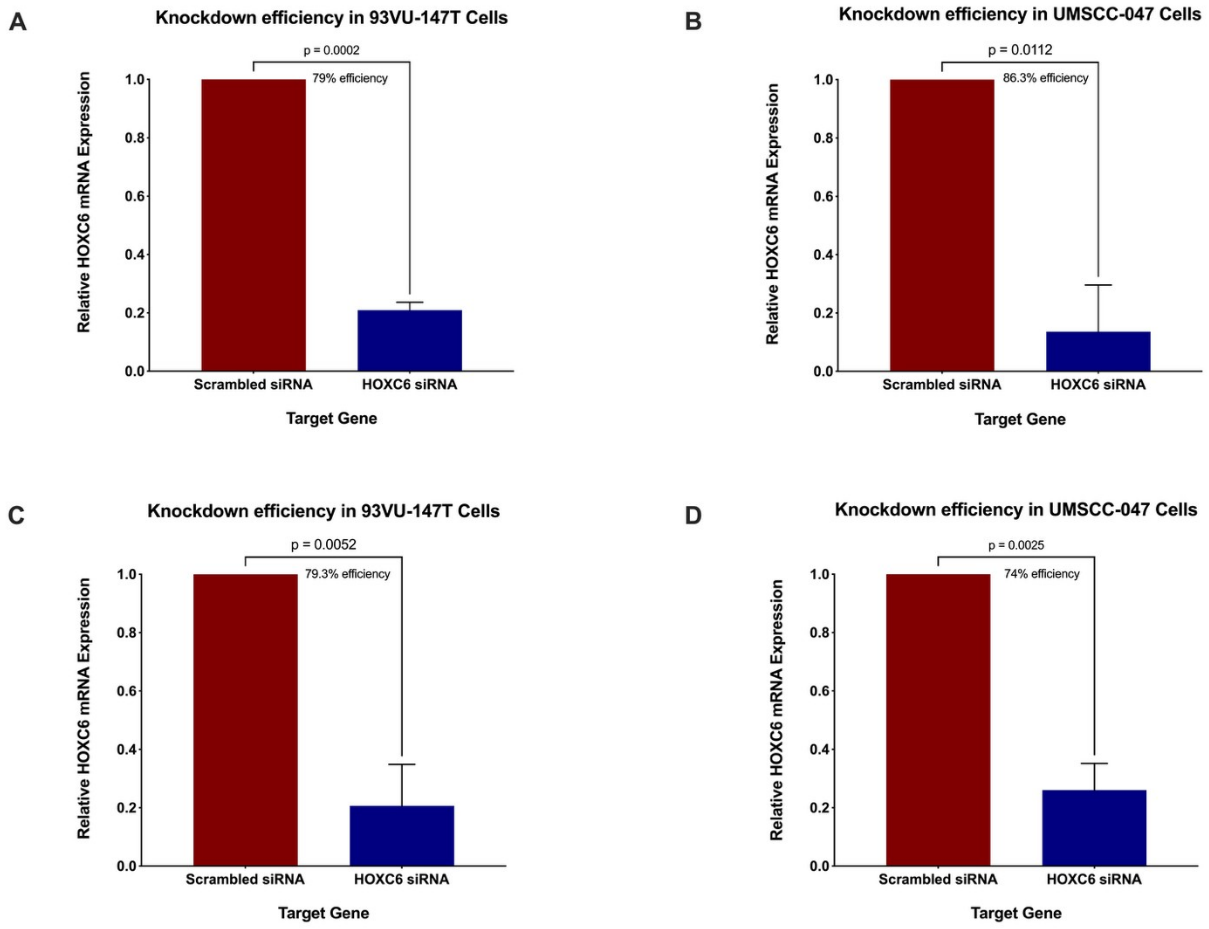

Supplementary Figure 4 (A-D). Relative expression of HOXC6 after transient knockdown of HOXC6 using siRNA specific to HOXC6 and the non-targeting siRNA for the (A and B) Cell Proliferation and (C and D) Wound Healing Assays. qRT-PCR was performed in the HNSCC cell lines **(A and C)** 93VU-147 T and **(B and D)** UMSCC-047 cells to analyze HOXC6 relative expression normalized to an internal control gene (β-actin). Relative expression values are expressed as fold changes; values less than one indicate downregulation (knockdown). For the knockdown study, HOXC6 was silenced for 72 hours and is presented as a bar graph for qRT-PCR data. Three independent experiments were performed in triplicate, and the data in the graph represent mean values of one representative experiment. The error bar represents the standard deviation (S.D.); the scale is on the left side. The percentage knockdown efficiency, calculated from qRT-PCR data, is indicated above each bar in the graph. Student's t-test was used to compare the target mRNA HOXC6 expression between scrambled siRNA (non-induction) and HOXC6 siRNA (induction) knockdown conditions. **$p < 0.01$

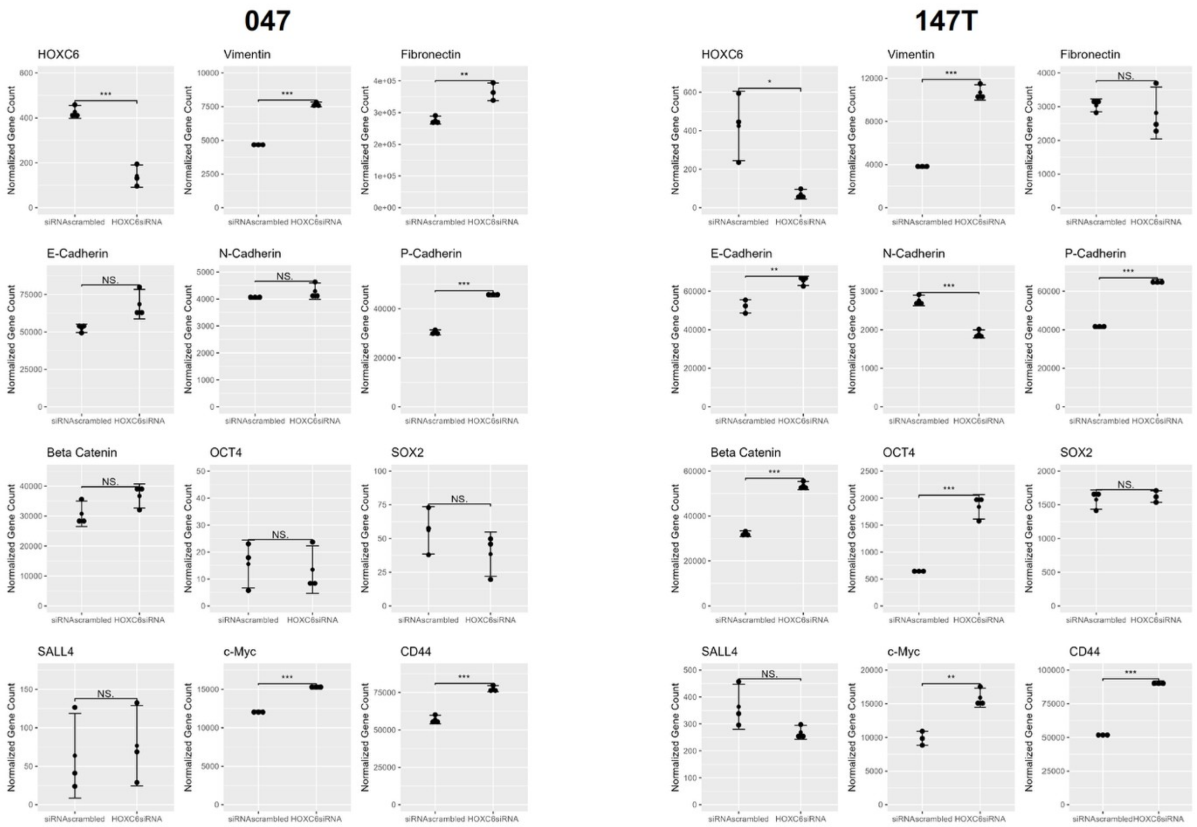

Supplementary Figure 5. HOXC6 silencing promotes EMT/stemness-like transcriptional changes in HSNCC cells. Normalized qPCR gene counts in UMSCC-47 (047) and 93VU-147T (147T) following control (scrambled siRNA) vs HOXC6 siRNA. HOXC6 is efficiently reduced in both lines. Upon HOXC6 silencing, E-cadherin decreases, while multiple mesenchymal markers (Vimentin, N-cadherin, P-cadherin, and fibronectin) increase. There was an increase in stemness markers (β-catenin, c-Myc, CD44, and OCT4). Significance: $p<0.05$; $p<0.01$; $p<0.001$; ns: not significant.

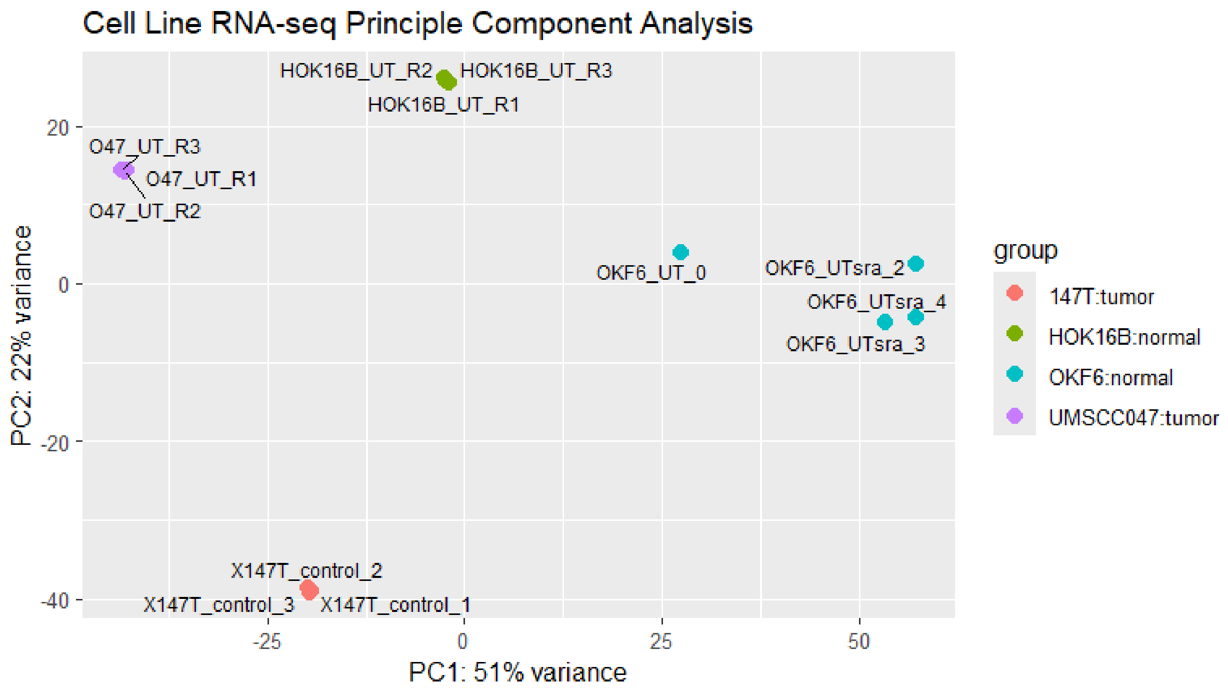

Supplementary Figure 6. Principal Component Analysis (PCA) of RNA-seq profiles from normal (OKF6 and HOK16B) and head and neck cancer (047 and 147T) cell lines. PCA was performed on variance-stabilized RNA-seq gene expression values to assess global transcriptional differences among the four cell line models. The first two principal components (PC1 and PC2) explain 51% and 22.8% of the variance, respectively. Normal oral keratinocyte lines (OKF6 and HOK16B) cluster distinctly from the head and neck cancer-derived lines (047 and 147T), reflecting clear separation based on transcriptional state. Biological replicates cluster tightly within each group, demonstrating high reproducibility. The tumor cell lines form separate clusters consistent with their unique molecular profiles, while OKF6 and HOK16B occupy distinct transcriptional spaces, reflecting baseline differences between the normal models. Colors denote group identity with each point representing an individual sample.

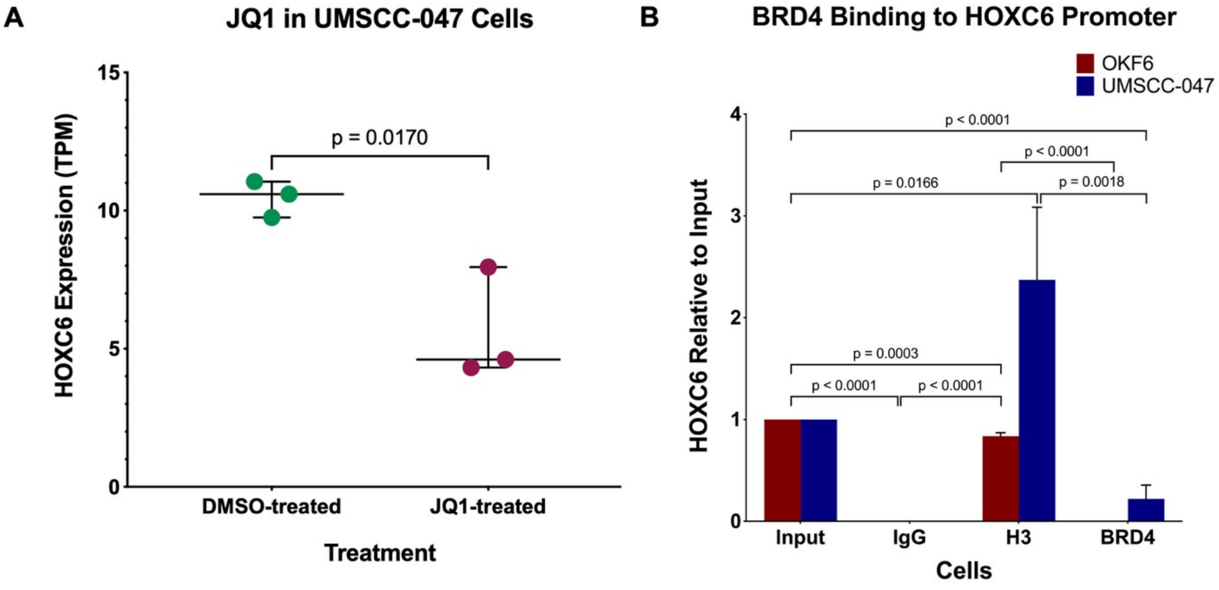

Supplementary Figure 7. BRD4 regulates HOXC6 transcription through promoter binding and BET inhibition. (A). HOXC6 expression was analyzed in UMSCC-047 cells that were treated with the BET inhibitor, JQ1, at a concentration of 500 nM for 72 hours, and DMSO-treated samples served as controls. HOXC6 expression levels were quantified using Transcripts Per Million (TPM) to ensure comparability across treatments. The x-axis represents different treatment conditions (DMSO and JQ1), while the y-axis denotes HOXC6 expression levels in TPM. The graph represents the mechanism of JQ1 action, where JQ1 inhibits BRD4 binding to acetylated histones, leading to reduced transcriptional activation. Data indicate that JQ1 treatment downregulates HOXC6 expression compared to DMSO-treated controls. Statistical significance was achieved at $p<0.05$. **(B).** ChIP-qPCR demonstrates BRD4 enrichment at the HOXC6 promoter in UMSCC-047 cells compared with normal oral keratinocytes (OKF6). Significant BRD4 binding was observed in UMSCC-047 cells relative to OKF6, confirming tumor-specific promoter occupancy by BRD4. Statistical significance was achieved at $p<0.05$.
